# Different hypothermic and cerebral perfusion strategies in extended arch replacement for acute type a aortic dissection: a retrospective comparative study

**DOI:** 10.1186/s13019-020-01284-y

**Published:** 2020-09-07

**Authors:** Song-Bo Dong, Jian-Xian Xiong, Kai Zhang, Jun Zheng, Shang-Dong Xu, Yong-Min Liu, Li-Zhong Sun, Xu-Dong Pan

**Affiliations:** 1 Department of Cardiovascular Surgery, Beijing Anzhen Hospital, Capital Medical University, and Beijing Institute of Heart Lung and Blood Vessel Diseases, Beijing, 100029 China; 2 Department of Cardiovascular Surgery, the first affiliated hospital of Gannan Medical University, Ganzhou, Jiangxi province China

**Keywords:** Acute type a aortic dissection, Total arch replacement, Moderate hypothermic circulatory arrest, Bilateral selective antegrade cerebral perfusion

## Abstract

**Background:**

The optimal hypothermic level in total arch replacement with stented elephant trunk implantation for acute type A aortic dissection (aTAAD) has not been established, and the superiority of unilateral or bilateral cerebral perfusion remains a controversial issue. Therefore, we evaluated the application of moderate hypothermic circulatory arrest (MHCA) with a core temperature of 29 °C and bilateral selective antegrade cerebral perfusion in aTAAD treated by total arch replacement with stented elephant trunk implantation.

**Methods:**

From July 2019 to January 2020, 25 aTAAD patients underwent total arch replacement with stented elephant trunk implantation via MHCA (29 °C) and bilateral selective antegrade cerebral perfusion (modified group). Thirty-six patients treated by the same procedure with MHCA (25 °C) and unilateral selective antegrade cerebral perfusion during this period were selected as controls.

**Results:**

There were no differences between the two groups of patients in terms of age, sex, incidence of hypertension, malperfusion, and pericardial effusion, although the incidence of cardiac tamponade was higher in the modified group (control 2.8%, modified 20%; *P* = 0.038). The lowest mean circulatory arrest temperature was 24.6 ± 0.9 °C in the control group, and 29 ± 0.8 °C in the modified group (*P* <  0.001). In-hospital mortality was 4.9% (3/61) for the entire cohort (control 8.3%, modified 0; *P* = 0.262). The incidence of permanent neurologic deficit was 4.9% (control 8.3%, modified 0; *P* = 0.262). There were no significant differences in the occurrence of temporary neurological deficit, renal failure, and paraplegia between groups. The rate of major adverse events in the modified group was lower (30.6% vs. 4%, *P* = 0.019). A shorter duration of ventilation and ICU stay was identified in the modified group, as well as a reduced volume of drainage within the first 48 h and red blood cell transfusion.

**Conclusions:**

The early results of MHCA (29 °C) and bilateral selective antegrade cerebral perfusion applied in total arch replacement with stented elephant trunk implantation for aTAAD were acceptable, providing similar inferior cerebral and visceral protection compared with that of the conventional strategy. A higher core temperature may account for the shorter duration of ventilation and ICU stay, as well as a reduced volume of drainage and red blood cell transfusion.

## Introduction

Acute type A aortic dissection (aTAAD), which is a catastrophic emergency, requires immediate surgical repair and is correlated with considerable risk of mortality and morbidity.

Since 2003, we have applied total arch replacement (TAR) with stented elephant trunk (SET) implantation by deep or moderate hypothermic circulatory arrest (MHCA) and unilateral selective antegrade cerebral perfusion (uSACP) via direct right axillary artery cannulation as our primary strategy for aTAAD in indicated patients with satisfactory results [1, 2].

Despite the improved perioperative results, some disadvantages exist in this standard protocol. We use core temperature of 25 °C as our target during distal arch anastomosis, which leads to extensive cooling and rewarming times, longer operation and cardiopulmonary bypass (CPB) times, platelet dysfunction, and inappropriate cerebral vasoconstriction. The ability of uSACP to afford adequate flow in both cerebral hemispheres is uncertain, especially in emergency cases with an incomplete Willis circle.

Bilateral selective antegrade cerebral perfusion (bSACP) assisted by a higher core temperature was recently attempted in aTAAD treated by TAR with SET implantation. Here, we conducted a retrospective comparative study to compare early outcomes of extended arch replacement using two different cerebral perfusion and thermal management strategies.

## Methods

This study was approved by the Ethics Committees of Beijing Anzhen Hospital, Capital Medical University and the First Affiliated Hospital of Gannan Medical University. Written informed consent was obtained from each patient.

### Patients

Between July 2019 and January 2020, 25 consecutive aTAAD patients underwent TAR with SET implantation by MHCA (29 °C) and bSACP in the two centers. This cohort of patients was defined as the modified group. Thirty-six aTAAD patients treated by the same TAR strategy via MHCA (25 °C) and uSACP during this period were selected as the control group. Patients with aTAAD were included in this study, with the exception of cases with persistent coma and hematochezia caused by severe malperfusion.

The mean age of the cohort was 50.1 ± 11.3 years, and 50 patients (82%) were male. Hypertension was the most prevalent comorbidity, being present in 49 patients (80.3%), followed by pericardial effusion in 29 cases (47.5%) and smoking in 26 cases (42.6%). Moderate or severe aortic insufficiency was confirmed in 18 cases (29.5%), malperfusion in 16 (26.2%), and cardiac tamponade in six (9.8%). Details of the clinical characteristics of the patients in both groups are summarized in Table 1. Aortic computed tomography angiography (CTA) and transthoracic echocardiography were performed routinely in the emergency department for confirmation of diagnosis, to evaluate the cardiac function, and to identify involved aortic valves and pericardial effusion.

**Table 1:** Preoperative clinical profiles

Variable	Control group (*n* = 36)	Modified group (*n* = 25)	*P* value
Age, years	49.8 ± 11	50.6 ± 12	0.807
Male	30 (83.3%)	20 (80%)	0.739
Hypertension	30 (83.3%)	19 (76%)	0.479
Smoking	16 (44.4%)	10 (40%)	0.730
Malperfusion	11 (30.6%)	5 (20%)	0.357
Pericardial effusion	20 (55.6%)	9 (36%)	0.133
Cardiac tamponade	1 (2.8%)	5 (20%)	0.038
Diabetes mellitus	1 (2.8%)	1 (4.0%)	1.000
Marfan syndrome	1 (2.8%)	1 (4.0%)	1.000
Myocardial infarction history	2 (5.6%)	1 (4.0%)	1.000
Previous cerebrovascular history	1 (2.8%)	2 (8.0%)	0.562
Previous aortic valve replacement	0	1 (4.0%)	0.410
Previous TEVAR for type B aortic dissection	0	2 (8.0%)	0.164
Dialysis	0	1 (4.0%)	0.410
Aortic Regurgitation (moderate or severe)	12 (33.3%)	6 (24%)	0.432
Ejection fraction	62.1 ± 5.3	60.7 ± 6.1	0.335

Data presented as mean ± SD, or percentages as appropriate

All survivors were monitored by clinic visits and phone calls, and the referring physician documented survival, reoperation and adverse events. Aortic CTA and echocardiography were recommended to evaluate the cardiac function and aorta annually.

### Definitions

In-hospital mortality was defined as death occurring during the hospital stay. Permanent neurologic deficit (PND) was defined as the presence of new focal (stroke) or global (coma) permanent neurological dysfunction confirmed by CT scanning. Temporary neurological deficit (TND) was defined as the presence of reversible postoperative confusion, delirium, agitation, or motor deficit. All symptoms were resolved before discharge after a normal CT scanning was obtained. The major adverse events (MAEs) included in-hospital mortality, PND, paraplegia, and new renal failure needing continuous renal replacement therapy (CRRT).

### Surgical technique

The method of TAR with SET implantation had been described in detail previously [2]. Indications for TAR included primary arch tear, involvement of arch vessels, Marfan syndrome and intimal intussusception in the arch.

In brief, the arterial line was bifurcated, one for axillary artery perfusion and the other for perfusion of a branch of the four-branched graft. After median sternotomy, the supra-aortic vessels were dissected individually.

Cooling was started immediately after the initiation of CPB. The proximal manipulations were carried out during the cooling period, including reinforcement of the detached commissures and ascending aorta or aortic root replacement. Once the target temperature was reached, all supra-aortic vessels were clamped and transected. The uSACP was initiated via the right axillary artery. The flow was adjusted to maintain a left radial artery pressure ≥ 20 mmHg. The stented elephant trunk (Cronus, MicroPort, China) was deployed in the true lumen of the descending aorta, and TAR was performed with a four-branched graft (Vascutek, Terumo, Japan). Once the distal anastomosis was completed, distal reperfusion was initiated. The left carotid artery was reconstructed first, and rewarming was then started. The ascending aorta was anastomosed to resume the myocardial perfusion, followed by the left subclavian artery, and finally the innominate artery.

In the modified group, the perfusing line prepared for the lower body was bifurcated again. Once the target temperature was reached, the innominate and left subclavian arteries were clamped, and the left carotid artery was cannulated to perfuse the left hemisphere. The left radial artery pressure was maintained at approximately 30 mmHg. We then performed the SET implantation and distal arch anastomosis. Subsequently, distal reperfusion was started via the third limb of the arterial line. The ascending aorta was anastomosed first, followed by the left subclavian artery reconstruction, the left carotid artery, and finally, the innominate artery.

### Statistical analysis

SPSS 19.0 (SPSS, Inc., Chicago, IL, USA) was applied for statistical analyses. The Kolmogorov–Smirnov test was used to assess normal or non-normal distribution of continuous data. Normally distributed continuous data were presented as means ± SD and assessed by Student’s *t*-test. Data showing non-normal distribution were presented as median and range interquartile, and the Mann–Whitney *U*-tests was performed. Chi-squared or Fisher’s exact tests were used in categorical variables.

## Results

There were no differences between the modified and control groups in terms of age, sex, incidence of hypertension, malperfusion, pericardial effusion, moderate or severe aortic regurgitation, and the preoperative cardiac function. The incidence of cardiac tamponade was higher in the modified group (20% vs. 2.8%, *P* = 0.038).

The operative data is summarized in Table 2. There were no differences between the groups in the incidence of concomitant procedures, such as aortic sinus repair, ascending aorta replacement, the Bentall procedure, bypass and CABG. Compared with the control group, the operation, CPB, cross-clamping, and lower body circulatory arrest time were shorter in the modified group (7 ± 0.8 h vs. 7.8 ± 1.2 h (*P* = 0.005); 174 ± 29.1 min vs. 218 ± 39 min (*P* <  0.001); 96.2 ± 20.4 min vs. 129.3 ± 26.4 min (*P* <  0.001); and 16 ± 4 min vs. 22.8 ± 4.9 min (*P* <  0.001), respectively). The perfusion pressure and cerebral perfusion flow were similar between the groups.

**Table 2:** Operative outcomes

Variable	Control group (*n* = 36)	Modified group (*n* = 25)	*P* value
Operation time, h	7.8 ± 1.2	7 ± 0.8	0.005
CPB time, min	218 ± 39	174 ± 29.1	< 0.001
Cross-clamp time, min	129.3 ± 26.4	96.2 ± 20.4	< 0.001
Lower body circulatory arrest time, min	22.8 ± 4.9	16 ± 4	< 0.001
Core temperature, °C	24.6 ± 0.9	29 ± 0.8	< 0.001
Pump pressure, mmHg	63 ± 14.2	65.4 ± 9.8	0.430
Cerebral perfusion flow ml/min	579.4 ± 166	571 ± 104.3	0.831
ml/kg·min	7.4 ± 2.0	7.8 ± 1.2	0.245
uSACP time, min	40 ± 7.7		
bSACP time, min		16 ± 4	
Aortic sinus repair	11 (30.6%)	9 (36%)	0.656
Ascending aorta replacement	25 (69.4%)	17 (68%)	0.905
Bentall	11 (30.6%)	8 (32%)	0.905
Extra-anatomic bypass	2 (5.6%)	0	0.508
CABG^※^	2 (5.6%)	1 (4.0%)	1.000

Data presented as mean ± SD, or percentages as appropriate

^※^*CABG*: coronary artery bypass grafting

The rates of early mortality and morbidity are presented in Table 3. Compared with the control group, the rate of MAE in the modified group was lower [30.6% (11/36) vs. 4% (1/25), *P* = 0.019]. There was no significant difference in the proportions of in-hospital mortality between the groups, with the deaths of three patients (8.3%) recorded in the control group and no deaths in the modified group (*P* = 0.262). Reasons for mortality were bilateral cerebral infarction, sudden cardiac arrest and septic shock (one patient for each).

**Table 3:** Postoperative results

Variable	Control group (*n* = 36)	Modified group (*n* = 25)	*P* value
MAEs^※^	11 (30.6%)	1 (4.0%)	0.019
In-hospital mortality	3 (8.3%)	0	0.262
PND	3 (8.3%)	0	0.262
TND	5 (13.9%)	2 (8.0%)	0.689
New renal failure with CRRT	5 (13.9%)	0	0.072
Paraplegia	2 (5.6%)	1 (4.0%)	1.000
Re-exploration for bleeding	2 (5.6%)	0	0.508
Tracheotomy	2 (5.6%)	1 (4.0%)	1.000
Ventilation time, h	29.8 (13.8, 66.4)	16 (12, 25)	0.038
ICU stay time, h	58.8 (21.6, 148.4)	34 (18.5, 50)	0.046
Chest tube drainage, ml/48 h	1020.6 ± 374	797.2 ± 180.1	0.004
RBC transfusion, unit^※^	5.5 (0.5, 10)	2 (0, 6.5)	0.045
without RBC transfusion	9 (25%)	12 (48%)	0.063

Data presented as mean ± SD, range interquartile or percentages as appropriate

^※^ MAEs: in-hospital mortality, *PND, * paraplegia, and new renal failure needing CRRT;

*RBC* red blood cell

Cerebral infarction occurred in three patients (8.3%) in the control group, while there were no cases of cerebral infarction in the modified group (*P* = 0.262). In addition to one death caused by bilateral massive cerebral infarction, two other patients suffered from this complication. The first patient, a 55-years-old male, experienced left hemiplegia due to right parietal lobe infarction 1 day after surgery. After rehabilitation therapy, his myodynamia of left side limbs was well recovered at 6 months follow-up. Apathy and left hemiplegia were noted in another 52-year-old male 2 h after surgery. The tracheal intubation was removed 27 h after the operation, and right hemisphere infarction was confirmed by subsequent CT scanning. He was transferred to a local rehabilitation center for functional recovery. At the six-month follow-up, he was able to walk slowly.

TND was observed in five patients (13.9%) in the control group and two patients (8.0%) in the modified group (*P* = 0.689).

Compared with the control group, the duration of mechanical ventilation was significantly shorter in the modified group [29.8 (13.8, 66.4) vs. 16 (12, 25), *P* = 0.038], as was the duration of the ICU stay [58.8 (21.6, 148.4) vs. 34 (18.5, 50), *P* = 0.046]. Compared with the control group, there was a lower volume of chest tube drainage within the first 48 h and red blood cell transfusion in the modified group [(1020.6 ± 374) vs. (797.2 ± 180.1), *P* = 0.004] and [5.5 (0.5, 10) vs. 2 (0, 6.5), *P* = 0.045], respectively. There were no significant differences in the incidence of new acute renal failure requiring CRRT, paraplegia, re-exploration for bleeding, and tracheotomy between the two groups.

Thirty-three patients in the control group and 25 patients in the modified group were discharged from the hospital and followed up to August 2020. Follow-up was completed for all survivors, with an average of 10 ± 2 months (median, 9; range, 7 to 13 months). No major cardiac and cerebral adverse events occurred in either of the groups during follow-up, and none of the patients needed further intervention.

## Discussion

Arch surgery may be mandatory to improve the prognosis of patients with aTAAD with arch involvement. The first case of aortic arch replacement with hypothermic circulatory arrest was performed in Bologna by Pierangeli in 1974 [3]. However, some drawbacks of this approach were been reported, including prolonged CPB time, higher incidence of neurologic dysfunction, limited safe time of distal circulatory arrest, and clotting disturbances [4]. To alleviate the side-effects of DHCA, a composite strategy incorporating the hypothermic circulatory arrest and selective cerebral perfusion was been proposed. This led to an apparent consensus that different levels of MHCA assisted by continuous selective cerebral perfusion was the key protocol for open arch repair [5–7].

In fact, the core temperature during arch surgery increased gradually with an acceptable postoperative mortality and morbidity [2, 8–10]. However, the optimal hypothermic level in TAR with SET implantation remained a controversial topic.

Since July 2019, we have attempted a warmer temperature in TAR with SET implantation for aTAAD in indicated patients. We found that there were no significant differences between the control and modified groups in terms of operative mortality, postoperative neurologic deficit, and visceral injury, and no deaths, PND or new renal failure needing CRRT occurred in the modified group.

The in-hospital mortality in the entire cohort was 4.9% (3/61), and all deaths occurred in the control group. This result was comparable with our previous reports [1, 2, 11, 12]. We believed the modified surgical techniques and evolving perfusion and cerebral protection strategies accounted for this. However, it should be noted that the characteristics of patients in this study may have contributed to the acceptable results. First, the mean age in all patients was 50.1 ± 11.3 years, which was almost 10 years younger than the European series of aTAAD treated by frozen elephant trunk [13, 14]. Second, the rates of preoperative comorbidities were low. Third, natural selection was inevitable. Many patients were transferred from hundreds of miles away, and may die on the way to hospital.

Neurologic deficit was one of the main parameters used to evaluate arch surgery. The TND rate was 8.0% (2/25) in the modified group, and 13.9% (5/36) in the control group, although this difference did not reach the level of statistical significance.

The incidence of PND was 4.9% (3/61), and all three cases occurred in the control group (8.3% vs. 0, *P* = 0.262). This result was comparable with other published series focusing on patients who underwent frozen elephant trunk, in which the rate of PND ranged from 8.8 to 13% [15, 16].

In a study of the application of uSACP and bSACP for varying extents of arch replacement in 307 aTAAD patients performed by Norton et al. [17], in which risk factors for postoperative stroke were analyzed. It was observed that the type of SACP, HCA time, the extent of arch replacement were not significantly associated with new-onset postoperative stroke. Several factors may be related this and the mechanisms of PND require further investigation.

The right axillary artery was routinely used for CPB and SACP, which rarely involved dissection and atherosclerosis, and was associated with lower embolic stroke rates [18]. The blood flow was retrograde from the cannulating site to the arch, which may protect against embolic events in the right hemisphere during proximal aortic manipulations. In addition, the atherosclerotic plaques were always located at the base of the supra-arch vessels, which became the source of the embolus during arch manipulations. We performed isolated anastomosis of supra-aortic vessels with respective graft branches after clamping the vessels, and the arch roof with potential atheromasia was excised totally.

The relative benefits of uSACP compared with bSACP as cerebral perfusion strategies remained undetermined. In a comparative cerebral protection study from the German Registry for aTAAD [19], including 1081 patients from 44 cardiac centers, it was found that the early neurologic outcomes with uSACP and bSACP were equivalent. A meta-analysis performed by Malvindi et al., consisting of 17 studies with 2949 bSACPs and 599 uSACPs, concluded that bSACP allowed for longer SACP time, with increasing safety once the SACP time exceeded 40 to 50 min [20]. In a study by Tong et al. focusing on the clinical effect of bSACP and uSACP in TAR [21], no significant difference was identified with regard to 30-day mortality and postoperative PND. The four-branched graft anastomosed to the left carotid artery was used for left hemisphere perfusion, and they concluded that this approach avoided the risk of embolic injury by cannulating the left carotid artery.

We concluded that the bSACP represented a more physiologic perfusion, and contributed to a reduced risk of left hemisphere hypoperfusion in cases with an incomplete Willis circle. A flexible arterial cannula, designed for aortic cannulation in children with a diameter of 16-18F, was used to cannulate the left carotid artery. Great attention must be paid to identify the true lumen. The intima was always edematous and fragile, requiring cannulation with extreme caution. The absence of PND in the modified group confirmed that no embolic event related to cannulation in left carotid artery occurred in these patients.

Although the optimal hypothermic level for organ protection in arch surgery remains to be established, the application of aggressive temperature has been reported in recent years [8, 22]. The use of moderate-to-mild levels of hypothermia ameliorates the adverse effects of deep hypothermia, including longer CPB times, coagulopathy, and other complications that result from temperature-related systemic vasoconstriction [23].

In a study of the clinical effect of different levels of systemic hypothermia during arch repair in aTAAD patients, Zierer et al. [8] found that SACP with MHCA (30 °C) was a safe tool for brain protection and helped to reduce hypothermia-related side-effects in comparison with the DHCA group. In another study [24], 1002 patients undergoing hemiarch (684, 68%) or total arch (318, 32%) replacement via SACP with a mean core temperature of 30 °C were analyzed. The rates of early mortality, paraplegia and acute renal failure requiring hemofiltration were 5, 0.3, and 4% respectively. It was concluded that a slightly higher cerebral perfusion pressure in combination with warm antegrade cerebral perfusion might allow for an important collateral flow from the brain to the spinal cord that is evidenced as a substantial backflow of blood from the descending aorta requiring continuous suction during open distal anastomosis.

In a comparative study performed by Shen et al. [25], the arch-first technique was applied for open total arch replacement in aTAAD patients under moderate systemic hypothermia (27.4 °C) and antegrade cerebral perfusion. Compared with the control group treated with DHCA (24.2 °C), the arch-first group had a significantly lower rate of distal organ complications (renal failure, hepatic failure and paraplegia).

The damaging effects of hypothermia on cerebrovascular autoregulation should not be ignored. Several animal studies have shown that cold cerebral perfusion can lead to neurologic injury due to its negative effects on cerebral autoregulation [26, 27], whereas mild systemic hypothermia preserved cerebral autoregulation and favored equal distribution of flow in the brain [28].

In our study, the occurrence of visceral injury in the setting of a higher core temperature was not noted. In the modified group, neither new renal failure needing CRRT nor hepatic and gastrointestinal injury were reported. One patient developed delayed paraplegia 3 days after surgery, and he recovered well 7 days later after cerebrospinal fluid drainage. The critical intercostal artery originated from the false lumen, and complete thrombus formation was observed in postoperative CTA performed 4 days after surgery. We considered the cause of delayed paraplegia may be related to rapid thrombus formation in the false lumen and inadequate collateral circulation in the acute phase, rather than the thermal strategy or distal open anastomosis.

Furthermore, the satisfactory visceral protection may be partially explained by the shorter lower body circulatory arrest time. In a study of open arch repair with moderate-to-mild (≥28 °C) systemic hypothermia performed by Ahmad et al. [6], an acceptable organ protection effect was achieved with a mean lower body circulatory arrest time of 46 ± 8 min in a cohort of patients with a mean age of 68 ± 16 years. In our study, the mean time of open distal arch anastomosis was 16 ± 4 min in the modified group. The distal dissected arch was sandwiched by the four-branched graft and the unstented part of the SET, and additional reinforced sutures were not usually needed. Therefore, distal reperfusion with approximate flow and pressure can be initiated once the distal anastomosis is completed.

In our previous study of 456 aTAAD patients treated by the same procedure with MHCA (25 °C) and uSACP, the mortality, incidence of PND, renal failure, and spinal cord injury were 8.1, 4.8, 4.4, and 2.4%, respectively [11]. The early postoperative outcomes were comparable to the results obtained in this study. We speculated that the effect of visceral protection using a higher core temperature was not inferior to that achieved by traditional thermal management strategy in the setting of TAR and SET implantation for aTAAD.

In this series, the duration of the operation, CPB, ventilation, and ICU stay were significantly lower in the modified group compared with that of the control group. We regarded cannulation of the left carotid artery as a convenient manipulation that costed only a small amount of time. The differences observed with respect to the duration of the operation, CPB, ventilation, and ICU stay may be explained to a large extent by the difference in hypothermia level. The clotting disorders related to prolonged CPB and low level MHCA were alleviated, which was confirmed by the significantly reduced volume of chest tube drainage in the first 48 h and RBC transfusion in the modified group.

### Study limitations

Certain limitations of this study should be noted. A retrospective comparative study cannot provide the same validity of evidence as a prospective study design. Although the comparable baseline characteristics in both groups, the existence of selection bias cannot be excluded.

In this study, 25 aTAAD patients were treated by two surgeons in modified group, and 36 patients were treated by another two surgeons in the control group. Although all four surgeons were experienced, the differences in operative data relating to the different surgeons treating the two groups may be magnified in the context of a limited sample.

Due to its small sample and retrospective nature, the statistical significance of our results require further confirmation in well-designed trials with larger sample sizes.

## Conclusions

In this study, the strategy of MHCA with a higher core temperature and bSACP was used for TAR and SET implantation in 25 indicated aTAAD patients at two centers, and satisfactory cerebral and visceral protection were achieved. There were no significant differences between the two groups in terms of in-hospital mortality and incidence of PND, with no deaths or cases of PND in the modified group. The higher core temperature may explain, in part, the shorter ventilation and ICU stay time in addition to a reduced volume of chest tube drainage during the first 48 h and RBC transfusion.

## Data Availability

The datasets used or analyzed in the study are available from the corresponding author on reasonable request.

## Abbreviations

aTAAD: Acute type A aortic dissection
DHCA: Deep hypothermic circulatory arrest
MHCA: Moderate hypothermic circulatory arrest
TAR: Total arch replacement
SET: Stented elephant trunk
uSACP: Unilateral selective antegrade cerebral perfusion
bSACP: Bilateral selective antegrade cerebral perfusion
CPB: Cardiopulmonary bypass
PND: Permanent neurologic deficit
TND: Temporary neurological deficit
MAEs: Major adverse events
CRRT: Continuous renal replacement therapy
